# Qualitative modification and development of patient- and caregiver-reported outcome measures for iron chelation therapy

**DOI:** 10.1186/s12955-017-0702-0

**Published:** 2017-06-23

**Authors:** Erica G. Horodniceanu, Vasudha Bal, Harman Dhatt, John A. Carter, Vicky Huang, Kathryn Lasch

**Affiliations:** 1 Pharmerit International, 4350 East West Highway, Suite 1110, Bethesda, MD 20814 USA; 2 Novartis Pharmaceuticals Corporation, One Health Plaza, East Hanover, NJ 07936 USA; 3 Pharmerit International, 300 Washington Street, Suite 410, Newton, MA 02458 USA

**Keywords:** Patient-reported outcomes, Observer-reported outcomes, Qualitative research, Iron chelation therapy, Cognitive debriefing, Instrument

## Abstract

**Background:**

Compliance, palatability, gastrointestinal (GI) symptom, and treatment satisfaction patient- and observer-reported outcome (PRO, ObsRO) measures were developed/modified for patients with transfusion-dependent anemias or myelodysplastic syndrome (MDS) requiring iron chelation therapy (ICT).

**Methods:**

This qualitative cross-sectional observational study used grounded theory data collection and analysis methods and followed PRO/ObsRO development industry guidance. Patients and caregivers of patients with transfusion-dependent anemias or MDS were individually interviewed face-to-face to cognitively debrief the Compliance, Palatability, GI Symptom Diary, and Modified Satisfaction with Iron Chelation Therapy (SICT) instruments presented electronically. Interviews were conducted in sets. Interviews began open-endedly to spontaneously elicit ICT experiences. Item modifications were debriefed during the later interviews. Interviews were audio recorded, transcribed, and coded. Data was analyzed using ATLAS.ti qualitative research software.

**Results:**

Twenty-one interviews were completed (Set 1: 5 patients, 6 caregivers; Set 2: 6 patients, 4 caregivers) in 6 US cities. Mean age was 43 years for patients and 9 years for children of caregivers. Conditions requiring ICT use across groups included transfusion-dependent anemias (85.7%) and MDS (14.3%). Concepts spontaneously reported were consistent with instruments debriefed. Interview analysis resulted in PRO and ObsRO versions of each instrument: Compliance (2 items), Palatability (4 items), GI Symptom Diary (6 items), and Modified SICT (PRO = 13, ObsRO = 17 items).

**Conclusion:**

Qualitative research data from cognitive interviews supports the content validity and relevance of the instruments developed/modified. Quantitative validation of these PRO and ObsRO measures is needed testing for validity, reliability, and responsiveness for future research use with new formulations of oral ICT.

## Background

Iron overload refers to the accumulation of excess plasma and cellular iron, and can occur in patients requiring frequent red blood cell transfusions [1, 2]. Typically occurring after 10–20 transfusions [3], iron overload is of particular concern for patients with transfusion-dependent anemias or myelodysplastic syndrome (MDS) [4]. In the United States alone, there are approximately 85,000 people with sickle cell disease (SCD) [5], 60,000 with MDS [6, 7], and 10,000 with beta-thalassemia [8]. It has been estimated that up to 20% of persons with SCD [9], 40% with MDS [10], and an undefined proportion with thalassemia are dependent on regular transfusions and are therefore susceptible to cardiac, hepatic, and endocrine morbidities caused by iron overload [1, 2, 9].

Because the human body has no regulated mechanism to excrete excess iron [2], persons with these conditions rely on iron chelation therapy (ICT) to remove excess iron and prevent iron accumulation, thus minimizing the clinical complications of iron overload, improving quality of life (QoL), and increasing life expectancy [1, 11, 12]. While life-preserving, ICT such as parenterally administered deferoxamine has been associated with significant deficits in QoL and consequently suboptimal treatment compliance [13–15]. An orally administered formulation, deferasirox (DFX), is now the most commonly used ICT [13, 16] and offers relatively better tolerability and convenience and thus greater treatment satisfaction and compliance versus deferoxamine [16–18]. However, DFX has restrictive administration instructions (eg, take on an empty stomach at least 30 min before food; disperse tablets by stirring in water or juice) [19] and known associations with suboptimal palatability, tolerability, and gastrointestinal (GI) side effects [19, 20], which may negatively impact compliance [20]. This direct relationship between treatment satisfaction and compliance among ICT and chronically-treated patients has been demonstrated previously [21–23], as has the relationship between perceived burden of ICT and poor compliance [22].

Poor compliance due to lack of palatability, tolerability, or QoL negatively impacted by side effects has long-term health implications given that inadequate treatment of chronic iron overload can lead to severe organ damage, particularly to the heart [24] and liver [25], and death [12]. To mitigate suboptimal DFX compliance, researchers have assessed an alternative, more flexible dosing and administration [20]. Clinical trials are underway to assess the associated compliance, palatability, GI symptom, and treatment satisfaction effects of the new DFX formulations. Valid, reliable assessment of associated treatment effects requires the development of new outcome measures that reflect ICT dosing and administration strategies that were not proposed at the time currently validated instruments were developed; yet no such instruments exist. As a result, this study was conducted to develop patient- and observer-reported outcome (PRO and ObsRO) measures needed in forthcoming DFX clinical trials.

## Methods

### Study design

A qualitative cross-sectional observational study protocol was developed in accordance with grounded theory [26–28] and approved by a central institutional review board (IRB) (Liberty IRB, Deland, FL). The protocol was also consistent with best practices for development and modification of PRO instruments and their implementation in electronic format [29], with the Food and Drug Administration’s Guidance to Industry for developing PRO instruments in support of labeling claims [30], and with guidance from professional organizations for establishing the content validity of newly developed and modified PRO instruments [31–33]. PRO refers to assessments based on direct responses from patients themselves, whereas ObsRO refers to assessments by a person other than the patient who is not a health professional and who is able to regularly observe and report on a patient’s health (eg, parent, spouse, or other caregiver). Observer reports are used for patients who are not able to self-report (eg, small children) [34].

Subjects were patients aged 10 years or older with transfusion-dependent anemias or MDS, or caregivers of patients aged 2 to 9 years with transfusion-dependent anemias. Inclusion and exclusion criteria purposefully mirrored those to be used in upcoming clinical trials. Inclusion criteria for patients were: males and females aged 10 years or older with transfusion-dependent anemias or MDS requiring frequent transfusions resulting in iron overload; previously or currently on oral ICT; history of transfusion of at least 20 packed red blood cell units; able to read, write, speak, and understand English; and provide informed consent. For pediatric patients, consent was obtained from parent(s) or legal guardian. Exclusion criteria for patients were: comorbidities that could affect item responses and/or increase medical risk (eg, GI conditions or diseases, such as ulcerative diseases, uncontrolled nausea, vomiting, diarrhea, malabsorption syndrome, or small bowel resection; psychiatric or addictive disorders); history of malignancy of any organ system treated or untreated within the past 5 years with the exception of localized basal cell carcinoma of the skin; currently participating in another clinical trial or receiving an investigational drug; history of non-compliance; or unable/unwilling to provide informed consent. Participating caregivers were also required to be able to read, write, speak, and understand English, and provide informed consent themselves; children (aged 2 to 9 years) of the participating caregivers were required to meet all other inclusion and exclusion criteria as described for patients. Subjects were recruited from US cities by 2 commercial patient recruitment agencies.

### Instrument development and selection

The Satisfaction with Iron Chelation Therapy (SICT) instrument was previously developed to assess patient-reported satisfaction and compliance with DFX dispersible tablets among patients with transfusion-dependent anemias or MDS and was validated in an open-label, single-arm, multicenter trial evaluating the efficacy and safety of DFX in patients with transfusion-dependent anemias and MDS requiring treatment for iron overload [21]. The SICT was originally developed by Novartis Pharmaceuticals as a PRO to assess satisfaction and compliance with DFX dispersible tablets [21]. The original 28-item SICT includes 8 domains: satisfaction with ICT effectiveness, safety/side effects, ICT convenience, costs, overall satisfaction, ICT impact on daily life, patient compliance, and ICT preferences. Given its previous validation and applicability here, modification of the SICT for new formulations was considered appropriate.

In addition to the SICT, new formulations required measures of GI symptomology, compliance, and palatability in order to compare the novel DFX preparations to the DFX dispersible tablet. Potential items and response options for these domains were initially provided by the study sponsor, Novartis Pharmaceuticals.

The study team reviewed the original SICT and draft Compliance, Palatability, and GI Symptom Diary items. This preliminary assessment served to remove items that were redundant or associated with concepts not appropriate to the approved or exploratory modes of DFX administration (eg, items pertaining to non-oral administration), and to revise wording of existing items deemed suitable for inclusion but for which relevance, readability, and comprehensibility could be improved. The resulting items were then modified to serve as potential ObsRO measure items for use with caregivers of children aged 2 to 9 years. Use of ObsRO measures has been recommended by an International Society for Pharmacoeconomics and Outcomes Research (ISPOR) Task Force for patients who are too young to report for themselves [35]. However, recommendations state that items should only assess events or behaviors that can be directly observed, without interpretation or inference by the observer [35].

### Patient and caregiver interviews

Trained interviewers conducted 21 in-person interviews according to pre-specified cognitive interview guides. The interviews, which were digitally recorded with subjects’ consent for the purpose of transcription, consisted of open-ended and structured components, conducted in that order. Interviews were conducted using the LogPad® electronic interface (provided by PHT Corporation; Boston, MA). Subjects were asked questions about the device use to assess the understanding and usability of the instruments in an electronic format.

The open-ended component elicited subjects’ experiences with ICT. During this concept elicitation portion of the interview, patients were asked, “Can you please describe for me what it is like for you to take medicine for iron overload?” When a symptom was mentioned, this initial question was followed by probes as needed regarding the frequency, duration, and bother of each symptom spontaneously reported to obtain further information. Similarly, caregivers of patients were asked, “Can you please describe for me what it is like for your child to take medicine for iron overload?” followed by probes on symptoms as needed. Caregivers were also asked, “Can you please describe for me what it is like for YOU for your child to take medicine for iron overload?” By design, interviewers gave an initial prompt but did not otherwise interject, except when clarification was needed to further explain subjects’ descriptions. The structured interview component included cognitive debriefing of the 4 previously described instruments to assess comprehensibility and relevance of the recall periods, instructions, items, and response options.

Using the “think-aloud” method [28], subjects described the meaning of the instruments’ content and the rationale for and process by which each item response was chosen. Note that items were debriefed concurrently rather than retrospectively [28]. Comprehension of scaled responses was assessed by prompting subjects to describe their interpretation of the highest and lowest responses in relation to the chosen response.

At the conclusion of the interview, subjects completed a demographic information form and were compensated $150 for their participation. Interviews were conducted and analyzed in iterative sets to allow evidence-based item modification before subsequent interviews.

### Analysis

All study data were de-identified and demographic and clinical data were analyzed descriptively. Analysis of the qualitative interviews was consistent with grounded methodology [26–28] for the responses to the open-ended question. Concepts spontaneously reported by patients and caregivers were identified. The coding scheme indicated if a debriefed item was interpreted as intended, relevant, or required rewording, and whether item stems and/or their response options needed modification. Qualitative data from the cognitive interviews were analyzed using ATLAS.ti (ATLAS.ti, version 7.1.8, GmbH; Berlin, Germany). A code book was developed through a standard iterative process whereby text segments were coded through a consensus-based process. Each transcript was coded independently and then reviewed by a senior study team member. Coding discrepancies and addition of new codes were harmonized among the study team. Conceptually equivalent codes that were applied to the open-ended question were merged.

## Results

### Demographic and clinical characteristics

Twenty-one subjects were interviewed in 6 US cities (Baltimore, MD; Boston, MA; Chicago, IL; Los Angeles, CA; New Orleans, LA; and St. Louis, MO). Version 1 of each instrument was debriefed with 5 patients and 6 caregivers; Version 2 with 6 patients and 4 caregivers. The majority of patients were male (72.7%) while the majority of caregivers were female (90.0%); caregivers included parents, grandparents, and aunts. Stratified subject characteristics are presented in Table 1.

**Table 1:** Demographic and clinical characteristics of patients, caregivers, and children of caregivers

	Patients (*N* = 11)	Caregivers (*N* = 10)	Children of Caregivers (*N* = 10)
Age, mean (range), y	43.25 (14.12–80.94)	47.63 (34.96–65.13)	8.90 (1.56–16.81)
Sex, *n* (%)			
Male	8 (72.73)	1 (10.00)	4 (40.00)
Female	3 (27.27)	9 (90.00)	6 (60.00)
Relationship to child, *n* (%)			
Mother	N/A	3 (30.00)	N/A
Father	N/A	1 (10.00)	N/A
Stepmother	N/A	1 (10.00)	N/A
Aunt	N/A	3 (30.00)	N/A
Grandparent	N/A	2 (20.00)	N/A
Race, *n* (%)			
Asian	1 (9.09)	1 (10.00)	2 (20.00)
Black or African American	7 (63.64)	8 (80.00)	8 (80.00)
White	3 (27.27)	1 (10.00)	0 (0.00)
Highest education level, *n* (%)			
High school or GED or less	5 (45.45)	0 (0.00)	N/A
College/university or Some college/certification	5 (45.45)	7 (70.00)	N/A
Graduate or Other	1 (9.09)	3 (30.00)	N/A
Current school grade, *n* (%)			
No School or Preschool	N/A	N/A	2 (20.00)
Grade 1-Grade 5	N/A	N/A	7 (70.00)
Other	N/A	N/A	1 (10.00)
Employment status^a^, *n* (%)			
Employed full-time or part-time	2 (18.18)	7 (70.00)	N/A
Unemployed or Retired	5 (45.45)	2 (20.00)	N/A
Student only or Other	4 (36.36)	1 (10.00)	N/A
Condition/diagnosis, *n* (%)			
MDS	3 (27.27)	N/A	0 (0.00)
TDA-Sickle cell disease	5 (45.45)	N/A	8 (80.00)
TDA-Aplastic anemia	1 (9.09)	N/A	0 (0.00)
TDA-Myelofibrosis	1 (9.09)	N/A	0 (0.00)
TDA-Thalassemia	1 (9.09)	N/A	2 (20.00)
Years with diagnosis^b^, mean (range)	11.67 (0.63–25.08)	N/A	7.15 (1.00–14.47)

Abbreviations: GED, general educational development; MDS, myelodysplastic syndrome; N/A, not applicable; TDA, transfusion-dependent anemias

^a^Employment status - “homemaker” was counted as “other”

^b^Years with diagnosis - (A) For those listing only the year of diagnosis, time with diagnosis was calculated from January 1 of the given year; (B) For those listing only the year and month of diagnosis, time with diagnosis was calculated from the first day of the given month; (C) For those listing only the year and/or year and month of diagnosis, where imputing as per notes A and B was not possible (ie, imputation would result in child’s diagnosis date occurring before the child’s date of birth), the child’s date of birth was imputed as the date of diagnosis

Twenty-one subjects were interviewed, including 11 patients for the development of the PRO instruments and 10 caregivers for the ObsRO instruments. Deviations from the protocol regarding the inclusion of caregivers occurred in 4 cases related to the caregiver’s child’s age at the time of the caregiver interview (1 child was younger than 2 years old, 3 children were older than 9 years old). One child was approximately 1.5 years of age at the time of the caregiver interview; however the caregiver provided pertinent data based on the child’s experience. Two of the children were near 10 years of age, but were too shy to participate themselves; their caregivers were willing to be interviewed instead. The last child was around 16 years of age; however, this child was treated between 2 to 9 years of age and the caregiver was able to provide relevant information from both the present and past. The exceptions were approved by the study principal investigator and the study sponsor.

Of the included patients and children of caregivers receiving ICT (*n* = 21), 14.3% (*n* = 3) had MDS and 85.7% (*n* = 18) had transfusion-dependent anemia. Of those with transfusion-dependent anemia (*n* = 18), 72.2% (*n* = 13) had sickle cell disease, 16.7% (*n* = 3) had thalassemia, 5.6% (*n* = 1) had myelofibrosis, and 5.6% (*n* = 1) had aplastic anemia. Overall, 57.1% (*n* = 12) of patients and children of caregivers combined were currently taking DFX. Commonly reported ongoing treatments for patients included DFX (73%), folic acid (27%), and hydroxyurea (27%). For children of caregivers, the most common medications were folic acid (70%), DFX (40%), hydroxyurea (40%), and penicillin (40%).

### Concepts spontaneously elicited

Many of the general concepts underlying the instruments (including satisfaction, compliance, and palatability with ICT therapy) were spontaneously elicited by the open-ended questions and were similar across interview sets for both PRO and ObsRO measures, suggesting their importance and relevance to patients and caregivers alike.

Patients and caregivers reported complying with their regimens for reducing iron overload, citing life-saving and health-promoting benefits. When compliance was problematic, patients and caregivers cited coping strategies in order to comply. However, 7 caregivers and 4 patients spontaneously reported “adherence” and/or issues related to compliance. Three patients described taking the medicine but it was questionable whether they were taking it as prescribed (ie, taken on an empty stomach and waiting 30 min after administration before eating, per DFX instructions). Another patient on DFX reported being compliant but responded, “It aggravates me that I have to take it every day and then wait a half an hour before I eat.” Caregivers noted that compliance was related to a child’s awareness of treatment benefits and medication palatability. For example, 1 caregiver reported that her child was more compliant after explaining the therapeutic benefits of ICT therapy. Another caregiver commented on how it would help her child to be “normal”, saying, “Yeah, she just says whatever’s going to make her better so she can be able to have her normal day and normal activities for the day.”

Caregivers also spontaneously noted the relationship between taste and compliance. For example, a caregiver reported, “When he was younger…he would spit it out a lot of times after I gave it to him…” Although caregivers mixed the medicine with drinks, taste was still a commonly reported issue for children. One caregiver noted the “hour-long challenges” to get her child to take DFX and reported that after trying different liquids to mix it with and “split dosing,” the child recognized “that it [taking the medication] was not an option.” Another caregiver of a child taking DFX stated, “We mix it with orange juice. And he complains about the taste. Otherwise... he takes it regularly. He doesn’t miss any dosage. The only complaint that he has is the taste.” Two caregivers whose children (4 and 10 years old, respectively) had thalassemia and were on DFX mentioned taste as a reason why their child did not fully comply with taking his or her medicine, stating, “…she liked chocolate milk. We mixed it in chocolate milk. And then she… – you know, she would take a sip and then just didn’t want it” and “Right now, it is the act of taking the medicine that he finds bothersome, in addition to – the main reason is the taste and having to take it. So if, you know, ... it was mixed in food, in my mind, it would be easier for him to take it.” One caregiver of a child on DFX found that as the child got older, it was easier to have her take the medicine, stating, “Put it in her juice, and I’d tell her that it was in there… she’s used to taking medicine, but she still doesn’t like it."

Patients also spontaneously mentioned dissatisfaction with the taste and texture of the medication. One patient, for example, described DFX as a “nasty medicine” and said one needed to “do a lot of stirring... You got to stir it up until it turns to a foam.” Another patient on DFX reported, “The only thing I’ve experienced that causes any trouble is when it doesn’t dissolve all the way, and then it’s not great.” When asked how often that was a problem he replied, “3 [out of 10 times] DFX does not dissolve.” Two patients reported being compliant with DFX, and described that someone might not take DFX as prescribed “because some people… may have a problem with the taste of the medication and swallowing it.”

Finally, 3 patients and 1 caregiver spontaneously reported GI symptoms due to ICT. A patient reported, “I believe it's coming from the medicine that I take for the iron overload. I have problems with sometime just running to the bathroom, because I have almost like diarrhea.” Another patient mentioned nausea and vomiting in addition to diarrhea, saying, “I get diarrhea and feels like I’m about to throw up… I don’t throw up every morning, but a loose bowel every morning.” Only 1 caregiver, whose child was on DFX, spontaneously mentioned stomach aches. The researchers assessed saturation and found that in latter interviews of the second set, no new concepts were introduced by patients or caregivers; therefore further interviews were unnecessary.

### Cognitive debriefing of instruments

Modifications made to each of the instruments (Compliance, Palatability, GI Symptom Diary, and Modified SICT) are described broadly below. An item tracking matrix (ITM) was used to document in detail all modifications to instructions, item stems, and response options for all 8 instruments (PRO and ObsRO versions for the 4 instruments) as well as the rationale for them. The ITM is available upon request to the corresponding author.

### Compliance

The Compliance instrument (for PRO and ObsRO) initially included instructions and 3 questions that assessed if a patient took his/her ICT treatment that day. No subject indicated difficulty understanding the instructions. Subsequent changes to the instructions were minor and semantic in nature. However, some had difficulty understanding an item that assessed if the ICT was taken with food on that particular day. As reported by the subjects (predominantly treated with DFX), this difficulty arose because DFX is directed to be taken without food. This item was removed from both the PRO and ObsRO instruments due to the difficulty in understanding and because a related concept was assessed in the Modified SICT. The finalized PRO and ObsRO Compliance instrument included 2 items on whether medication was taken that day and the time of day taken (Table 2).

**Table 2:** PRO and ObsRO Compliance Instruments

#	Item Stem^a^	Response Scale
Inst	The following questions are about the medicine you take for iron overload (too much iron in your body). Please read each one and answer by yourself. There are no right or wrong answers. All of your answers will remain confidential. Please answer each question about your medicine for iron overload **TODAY**:	N/A
1	Did you take your medicine for iron overload today?	Yes/No
2	What time did you take your medicine for iron overload today?	Time medication taken: __:__ AM / PM; Not applicable (did not take medicine for iron overload today)

Abbreviations: Inst, instructions; N/A, not applicable; ObsRO, observer-reported outcome; PRO, patient-reported outcome

^a^Instructions, items, and response options reflect exact wording used in the PRO measure. The ObsRO measure is identical, with the exception of replacing “the medicine you take”/“your medicine” with “the medicine your child takes”/“the medicine your child took” in the instructions, and “you take your” with “your child take his/her” in items 1 and 2

### Palatability

The PRO Palatability instrument was initially composed of instructions and 5 items to assess taste, texture (ie, feel), the ability to swallow the medication, and volume of liquid needed for administration. The item assessing the feeling of medicine inside one’s mouth (ie, texture) was deleted because interview feedback indicated that subjects could not differentiate “texture” in this situation from “taste”. Other changes to the PRO version were minor.

**Fig. 1:**
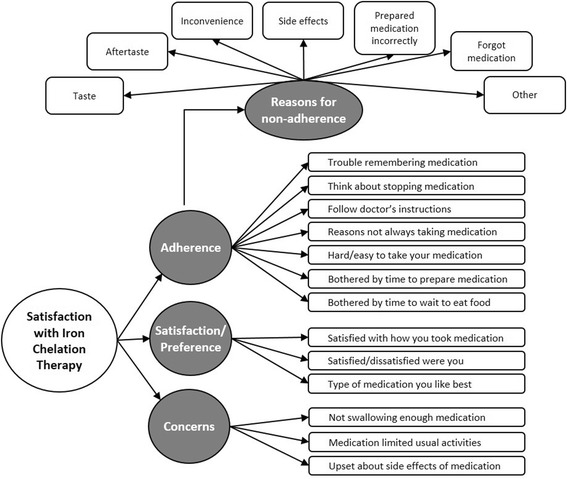
Conceptual Framework

The ObsRO Palatability instrument was initially composed of 8 items, although 3 items were presented as items branching to related items. Constructing a measure of palatability to be completed by caregivers was challenging given that the intent was to assess observable events. That is, the purpose was to assess palatability concepts as demonstrated by children, relayed through caregivers’ perceptions of their children’s reactions to taking the medication. To ensure that ObsRO items were observable, caregivers were asked to describe his/her child’s reaction to taste and feel. Caregiver responses were used to further revise both item stems and response scales in the finalized ObsRO measure. Exemplary quotes from caregivers’ descriptions of their child’s reactions related to items in the Palatability instrument are listed in Table 3. Given that caregivers consistently reported that their child would make a face in reaction to the medication taste and aftertaste, and suggested rewording supported this as well in the finalized ObsRO Palatability instrument, the response scale of very good to very bad now includes a corresponding faces scale. This change was made to make the caregiver report more closely mirror the observed reactions. The finalized PRO and ObsRO Palatability instruments included 4 items (Table 4). Figure 1 presents a conceptual framework linking satisfaction and reasons for non-compliance, compliance, and palatability.

**Table 3:** ObsRO Palatability Instrument – Supportive Caregiver Qualitative Data

Item (Version)	Concept	Quotation	Subject ID
Item 1 (Version 1)	Reaction to taste	“Did she like it or did she not like it? Did she like make a face or – when taking the medication?” INTERVIEWER: Make a face? Is that what she would do? “Mm-hmm [to indicate yes]. If it was nasty”	0205
Item 1 (Version 1)	Reaction to taste	“He just would frown”	0203
Item 1 (Version 2)	Reaction to taste	“He’d make a face. He would spit it out. He would, you know, say, agh, this is horrible. He would have something to say if it had any – especially if it had a bad taste to him.”	0206
Item 1 (Version 2)	Reaction to taste	“From facial expressions, say so – not want to take it”	0208
Item 2 (Version 1)	Reaction to feel	“If she gagged, did she throw up or did she, you know, spit up or make some type of frown face?”	0201
Item 4 (Version 1)	Reaction to taste after swallowing	“It couldn’t have been nasty, because she would have reached for something”	0201
Item 4 (Version 1)	Reaction to taste after swallowing	“Well, you can just see the reaction on her face. There would be a frown or a sound, you know? Reaction to the taste of the medicine’s where she makes a sound or a facial impression.”	0204
Item 4 (Version 1)	Reaction to taste after swallowing	“She would have made a face.”	0205
Item 4 (Version 1)	Reaction to taste after swallowing	“There’s times there is a little grit piece left. And she usually sticks her tongue out to show me or she’ll pull her lip down to show me, in which case I’ll usually give her a drink of water and have her swallow it.”	0601
Item 2 (Version 2)	Reaction to aftertaste	“Just facial expression or, you know, a comment or refusing to take it again”	0208
Item 2 (Version 2)	Reaction to aftertaste	“Making faces after she swallowed it, or you know, smacking her mouth”	0207

Abbreviation: ObsRO, observer-reported outcome

**Table 4:** PRO and ObsRO Palatability instruments

#	Item Stem^a^	Response Scale
PRO		
Inst	The following questions are about the medicine you take for iron overload (too much iron in your body). Please read each one and answer by yourself. There are no right or wrong answers. All of your answers will remain confidential. Please answer each question about your medicine for iron overload **TODAY**:	N/A
1	How did your medicine for iron overload taste today?	5-point scale: Very good, Good, Not good or bad, Bad, Very bad
2	Some medicines have an aftertaste (taste left in your mouth after you swallow). How was the aftertaste of your medicine for iron overload today?	
3	Using the answers listed below, which answer best describes what happened after you took your medicine for iron overload today?	4-point scale: Swallowed ALL of the medicine, Spat out SOME of the medicine, Spat out ALL of the medicine and swallowed none of it, Vomited within 30 min after swallowing the medicine
4	How would you describe the amount of liquid that you took with your medicine for iron overload today?	3-point scale: Not enough liquid, Just enough liquid, Too much liquid
ObsRO^b^		
1	Please choose the face that best describes your child’s reaction to the taste of the medicine for iron overload immediately after putting it in his/her mouth today.	5-point scale: Faces scale 1–5
2	Please choose the face that best describes your child’s reaction to the aftertaste of the medicine after swallowing his/her medicine for iron overload today.	

Abbreviations: Inst, instructions; N/A, not applicable; ObsRO, observer-reported outcome; PRO, patient-reported outcome

^a^Instructions, items, and response options reflect exact wording used in the PRO measure, unless otherwise specified. The ObsRO measure instructions were identical, with the exception of replacing “the medicine you take”/“your medicine” with “the medicine your child takes”/“the medicine your child took”. The ObsRO items 3 and 4 stem and response scale were identical to the PRO, with the exception of replacing “you took your” with “your child took his/her” in item 3, and “you took with your” with “your child took with his/her” in item 4

^b^Items 1 and 2 item stems and response scale for the ObsRO were different from the PRO

### GI symptom diary

The GI Symptom Diary assessed abdominal pain, nausea, vomiting, constipation, diarrhea, and bowel movements. Severity of the latter 2 symptoms were assessed by stool frequency and consistency in the initial instruments. Changes made to the PRO and ObsRO versions (each initially containing 8 items) were similar and largely semantic. Notable revisions included the descriptions of “abdominal pain” and “stools.” Subjects frequently referred to “abdominal pain” as “pain in the belly,” and the item was revised accordingly. Although the word “stools” was initially included to describe “bowel movements,” subjects stated that the word was unnecessary, and it was therefore deleted.

Response options between the PRO and ObsRO GI Symptom Diary instruments vary. PRO response options elicit a severity rating using a numeric rating scale from 0 (no symptom) to 10 (worst symptom) for the 5 symptoms. ObsRO responses elicit the frequency of the symptom using a Likert format (always, most of the time, sometimes, rarely, never). The researchers agreed that caregivers would not be able to rate the severity of these symptoms from the child’s perspective, but they would be more likely to directly observe and rate their frequency.

Also of note is that an item intended to assess the proportion of bowel movements in the past 24 h that were “unexpected bowel movements” or “accidents” was removed. Subjects did not interpret these expressions correctly and had difficulty understanding what might constitute an “unexpected” versus an “expected” bowel movement. The latter issue was most pronounced in caregivers of young children, for whom “accidents” were difficult to define or differentiate from urgency. The finalized PRO and ObsRO GI Symptom Diary measures contained 6 items each (Table 5).

**Table 5:** PRO and ObsRO GI Symptom Diary instruments

#	Item Stem^a^	Response Scale^b^
Inst PRO	Please read each symptom carefully. For each symptom, choose the number between 0 and 10 to rate how severe the symptom was **over the past 24 h (for example, from 8:00 AM yesterday to 8:00 AM today)**. Zero ‘0’ means you did not have this symptom and ‘10’ means it is the worst level of the symptom you can have. Please answer all of the following questions after taking your daily dose of medicine for iron overload.	N/A
Inst ObsRO	Please read each symptom carefully. Indicate how often your child had each symptom **over the past 24 h (for example, from 8:00 AM yesterday to 8:00 AM today).** Please answer all of the following questions after your child has taken his/her daily dose of medicine for iron overload.	N/A
1	Pain in your belly	11-point horizontal scale: 0 to 10 (0 = No Pain; 10 = Worst Pain)
2	Nausea (feeling like you might throw up)	11-point horizontal scale: 0 to 10 (0 = No Nausea; 10 = Worst Nausea)
3	Vomiting (throwing up)	11-point horizontal scale: 0 to 10 (0 = No Vomiting; 10 = Worst Vomiting)
4	Constipation	11-point horizontal scale: 0 to 10 (0 = No Constipation; 10 = Worst Constipation)
5	Diarrhea	11-point horizontal scale: 0 to 10 (0 = No Diarrhea; 10 = Worst Diarrhea)
6	How many bowel movements did you have in the past 24 h?	0 (none), 1, 2, 3, 4, 5–10, 11 or more

Abbreviations: Inst, instructions; N/A, not applicable; ObsRO, observer-reported outcome; PRO, patient-reported outcome

^a^Instructions, items, and response options reflect exact wording used in the PRO measure, unless otherwise specified. The ObsRO measure instructions and response options differed from that of the PRO, while item stems were identical, with the exception of replacing “your” with “his//her” in item 1, “you” with “he/she” in item 2, and “you” with “your child” in item 6

^b^In the ObsRO measure, the response scale for items 1–5 consisted of 5-point scale (Always, Most of the time, Sometimes, Rarely, Never) instead of the 11-point scale used in the PRO

### Modified SICT

Several items on the original SICT were considered irrelevant when comparing orally administered versus intravenously (IV) infused ICT and were therefore removed from the Modified SICT prior to the first set of interviews. For example, an item asking how frequently one’s ICT regimen limited evening or night-time activities was not included because this was determined to be a concern for IV administered ICT only. Further, the recall period was revised from “in the last four weeks” in the original SICT, to “in the past week” in the first set of interviews, and to “in the past week (past 7 days)” in the finalized Modified SICT.

Eleven items on the first PRO Modified SICT instrument were deleted from the original SICT due to lack of relevance to new ICT formulations and redundancy with other items. Other items were modified to provide colloquial wording. Five items were removed in the second interview/revision iteration, again owing to irrelevance to the current ICT formulations, redundancy, or comprehension issues. For example, an item asking subjects to weigh the relative benefits and undesirable effects of their ICT was deleted due to poor comprehension. The finalized PRO Modified SICT measure also included an item assessing the patient burden of meal restrictions, as subjects reported bother associated with these requirements. The finalized PRO Modified SICT measure contained 13 items (Table 6).

**Table 6:** PRO Modified SICT instrument

#	Item Stem	Response Scale
Inst	The following questions are about the medicine you take for iron overload (too much iron in your body) **in the past week (past 7 days)**. Please read each one and answer by yourself. There are no right or wrong answers. All of your answers will remain confidential. Choose only one answer.	N/A
1	Over the past week, how often did you feel worried that you were not swallowing enough of your medicine for iron overload?	5-point scale: Always, Most of the time, Sometimes, Rarely, Never
2	Over the past week, how often did you feel your medicine for iron overload limited your usual activities?	
3	Over the past week, how often did you feel upset about the side effects of your medicine for iron overload?	
4	Over the past week, how often did you have trouble remembering to take your medicine for iron overload?	
5	Over the past week, how often did you think about stopping taking your medicine for iron overload?	
6	Over the past week, how often did you follow your doctor’s instructions for taking your medicine for iron overload?	
7	What are the reasons that you did not always take your medicine for iron overload as instructed by your doctor? (Choose all that apply)	Taste, Aftertaste (taste left in your mouth after you swallow), Inconvenience (it’s a problem to take your medicine), Side effects, Prepared the medicine incorrectly, Forgot to take the medicine, Other______
8	Over the past week, how easy or hard was it for you to take your medicine for iron overload?	5-point scale: Very easy, Easy, Neither easy nor hard, Hard, Very hard
9	Over the past week, how bothered were you by the amount of time it took to you to prepare your medicine for iron overload?	5-point scale: Very bothered, Quite bothered, Moderately bothered, A little bothered, Not bothered at all
10	Over the past week, how bothered were you by the amount of time you had to wait to eat food after taking your medicine for iron overload?	
11	Medicines can be taken in many ways (for example, tablet dissolved in liquid, tablet swallowed whole, powder sprinkled on food, or injection). Over the past week, how satisfied or dissatisfied were you with how you took your medicine for iron overload?	5-point scale: Very satisfied, Satisfied, Neither satisfied nor dissatisfied, Dissatisfied, Very dissatisfied
12	Over the past week, how satisfied or dissatisfied were you with the medicine you took for iron overload?	
13	Which type of medicine for your iron overload do you like best?	Tablet to dissolve in liquid, Powder to sprinkle on food, Tablet to swallow, I don’t know

Abbreviations: Inst, instructions; N/A, not applicable; PRO, patient-reported outcome; SICT, Satisfaction with Iron Chelation Therapy

Where appropriate, the modifications described for the PRO Modified SICT were then applied to create an ObsRO Modified SICT. However, the purpose of the ObsRO measure was not only to assess patients’ satisfaction with ICT by proxy, but also caregivers’ own satisfaction with ICT. Therefore, the final 17-item ObsRO version is divided into 2 sections (Table 7).

**Table 7:** ObsRO Modified SICT instrument

#	Item Stem	Response Scale
Inst 1	The following questions are about the medicine your child takes for iron overload (too much iron in the body) **in the past week (past 7 days)**. Please read each one and answer by yourself. There are no right or wrong answers. All of your answers will remain confidential. Choose only one answer.	N/A
1	Over the past week, how often did your child’s medicine for iron overload limit his/her usual activities?	5-point scale: Always, Most of the time, Sometimes, Rarely, Never
2	Over the past week, how often was your child upset about the side effects of his/her medicine for iron overload?	
3	Over the past week, how often did your child take his/her medicine for iron overload?	
4	Over the past week, how often did your child express that he/she wanted to stop taking medicine for iron overload?	
5	Over the past week, how often did your child follow the doctor’s instructions for taking his/her medicine for iron overload?	
6	What are the reasons expressed by your child for not always taking his/her medicine for iron overload as instructed by the doctor? (Choose all that apply)	Taste, Aftertaste (taste left in your child’s mouth after swallowing his/her medicine), Inconvenience (for child), Prepared the medicine incorrectly, Other______
7	Over the past week, how easy or hard did your child tell you it was to take his/her medicine for iron overload?	5-point scale: Very easy, Easy, Neither easy nor hard, Hard, Very hard
8	Over the past week, how bothered did your child express that he/she was by the amount of time he/she had to wait to eat food after taking medicine for iron overload?	5-point scale: Very bothered, Quite bothered, Moderately bothered, A little bothered, Not bothered at all
9	Please choose the face that best describes how happy or unhappy your child appeared with his/her medicine for iron overload over the past week.	5-point scale: Faces scale 1–5
10	Which type of medicine did your child say he/she liked best?	Tablet to dissolve in liquid, Powder to sprinkle on food, Tablet to swallow, I don’t know
Inst 2	The following questions are about YOUR experiences with the medicine your child takes for iron overload **in the past week (past 7 days)**. Please read each one and answer by yourself. There are no right or wrong answers. All of your answers will remain confidential. Choose only one answer.	N/A
11	Over the past week, how often did you feel worried that your child was not swallowing enough of his/her medicine for iron overload?	5-point scale: Always, Most of the time, Sometimes, Rarely, Never
12	Over the past week, how often did you give your child his/her medicine for iron overload?	
13	Over the past week, how often did you think to stop giving your child his/her medicine for iron overload?	
14	Over the past week, how often did you follow the doctor’s instructions for giving your child his/her medicine for iron overload?	
15	What are the reasons that you did not always give your child his/her medicine for iron overload as instructed by the doctor? (Choose all that apply)	Child refused to take, Forgot to give the medicine, Inconvenient for you or your child, Side effects (for child), Did not prepare the medicine according to the doctor’s instructions, Did not give the full amount of the prepared medicine, Other________
16	Over the past week, how easy or hard was it for you to give your child his/her medicine for iron overload?	5-point scale: Very easy, Easy, Neither easy nor hard, Hard, Very hard
17	Over the past week, how bothered were you by the amount of time it took to prepare your child’s medicine for iron overload?	5-point scale: Very bothered, Quite bothered, Moderately bothered, A little bothered, Not bothered at all

Abbreviations: Inst, instructions; N/A, not applicable; ObsRO, observer-reported outcome; SICT, Satisfaction with Iron Chelation Therapy

## Discussion

Qualitative data from 21 patients and caregivers underpin the development of 3 new PRO and ObsRO measures, the modification of the SICT PRO measure, and the development of the Modified SICT ObsRO measure. The draft instruments included the 2-item Compliance PRO and ObsRO, 4-item Palatability PRO and ObsRO, 6-item GI Symptom Diary PRO and ObsRO, 13-item Modified SICT PRO, and 17-item Modified SICT ObsRO instruments.

The challenge of medication adherence, compliance, and persistence to the long-term treatment of chronic conditions, including iron overload, is well established [13–15, 36, 37]. Palatability has been recognized as a major factor in compliance to oral medication, especially for pediatric populations [38]. The World Health Organization, regulators, and consumer action groups have requested further research into pediatric treatment adherence and compliance [38], but there is limited evidence on the relationship between palatability and treatment compliance. While the satisfaction with dispersible DFX reportedly has been higher than with IV DFX, compliance with DFX remains suboptimal. For example, a study with a 1-year follow-up of pediatric SCD patients found that self-reported DFX compliance was 71%, but only 43% by pill count [39]. The results presented here suggest the importance of taste in taking and adhering to dispersible DFX.

This study also found that patients and/or caregivers of patients may discontinue or modify ICT treatment due to GI symptoms, suggesting the importance of including a measure of GI symptoms when taking the new formulations of DFX. Finally, this research allowed for the explication of the reasons for adherence and the development of a testable conceptual model that explicates important factors associated with adherence and satisfaction with dispersible DFX.

There are many strengths of this study, including representation from patients with several types of conditions that require treatment for iron overload, with diverse educational backgrounds, from different geographic locations, and using different strategies to treat or prevent iron overload. The study included a wide age range of subjects, with patients ranging from approximately 14 to 81 years of age, and children of caregivers ranging from approximately 2 to 17 years of age. Despite this wide age range and the diversity of study subjects, there was consistency in their experiences with ICT. In addition, rigorous data collection and analysis methods were applied during this instrument development exercise.

One limitation, however, is that all patients and caregivers were English-speaking and currently living in the United States. If these instruments are planned for use in international trials, it will be important in future research to note any differences by country. A further limitation is that this study was cross-sectional, at times prompting patients or caregivers to rely on memory when reporting prior use of medication. In addition, the sample size was small, but is considered adequate when saturation is achieved [40, 41].

## Conclusion

This study supports the content validity and relevance of the Compliance, Palatability, GI Symptom Diary, and Modified SICT instruments for patients with transfusion-dependent anemias or MDS requiring ICT based on qualitative research. Treatment-specific measures such as these may prove to be more valuable than generic treatment satisfaction measures to explain the relationships between satisfaction and compliance given that they directly measure important components of satisfaction with oral medications such as palatability and treatment side effects such as GI symptoms, which are a common concern with ICT. Routine use of these PRO and ObsRO measures in clinical research with new formulations of oral ICT awaits psychometric validation.

## Abbreviations

DFX: Deferasirox
GI: Gastrointestinal
ICT: Iron chelation therapy
IRB: Institutional review board
ISPOR: International Society for Pharmacoeconomics and Outcomes Research
ITM: Item tracking matrix
IV: Intravenous
MDS: Myelodysplastic syndrome
ObsRO: Observer-reported outcome
PRO: Patient-reported outcome
QoL: Quality of life
SCD: Sickle cell disease
SICT: Satisfaction with Iron Chelation Therapy
US: United States

## References

[1] Poggiali E, Cassinerio E, Zanaboni L, Cappellini MD (2012). An update on iron chelation therapy. Blood Transfus.

[2] Chalmers AW, Shammo JM (2016). Evaluation of a new tablet formulation of deferasirox to reduce chronic iron overload after long-term blood transfusions. Ther Clin Risk Manag.

[3] Andrews NC (1999). Disorders of iron metabolism. N Engl J Med.

[4] Hoffbrand AV, Taher A, Cappellini MD (2012). How I treat transfusional iron overload. Blood.

[5] Hassell KL (2010). Population estimates of sickle cell disease in the U.S. Am J Prev Med.

[6] Sekeres MA (2011). Epidemiology, natural history, and practice patterns of patients with myelodysplastic syndromes in 2010. J Natl Compr Cancer Netw.

[7] Ma X (2012). Epidemiology of myelodysplastic syndromes. Am J Med.

[8] Centers for Disease Control and Prevention. Diseases and Conditions: Thalassemia. https://www.cdc.gov/features/international-thalassemia/. Updated 05 May 2016. Accessed 18 Apr 2017.

[9] Wood JC (2008). Cardiac iron across different transfusion-dependent diseases. Blood Rev.

[10] Mitchell M, Gore SD, Zeidan AM (2013). Iron chelation therapy in myelodysplastic syndromes: where do we stand?. Expert Rev Hematol.

[11] Cappellini MD, Taher A (2009). Deferasirox (Exjade) for the treatment of iron overload. Acta Haematol.

[12] Delforge M, Selleslag D, Beguin Y, Triffet A, Mineur P, Theunissen K (2014). Adequate iron chelation therapy for at least six months improves survival in transfusion-dependent patients with lower risk myelodysplastic syndromes. Leuk Res.

[13] Porter J, Bowden DK, Economou M, Troncy J, Ganser A, Habr D, et al. Health-related quality of life, treatment satisfaction, adherence and persistence in beta-thalassemia and myelodysplastic syndrome patients with iron overload receiving deferasirox: results from the EPIC clinical trial. Anemia. 2012;2012:297641. 10.1155/2012/297641 PMC3424665 22924125

[14] Payne KA, Rofail D, Baladi JF, Viala M, Abetz L, Desrosiers MP (2008). Iron chelation therapy: clinical effectiveness, economic burden and quality of life in patients with iron overload. Adv Ther.

[15] Payne KA, Desrosiers MP, Caro JJ, Baladi JF, Lordan N, Proskorovsky I (2007). Clinical and economic burden of infused iron chelation therapy in the United States. Transfusion.

[16] Kwiatkowski JL (2011). Real-world use of iron chelators. Hematol Am Soc Hematol Educ Prog.

[17] Kwiatkowski JL, Kim HY, Thompson AA. Chelation choices and iron burden among patients with thalassemia in the 21st century: a report from the Thalassemia Clinical Research Network (TCRN) longitudinal cohort [abstract]. Blood. 2009;114:4056.

[18] Cappellini MD, Bejaoui M, Agaoglu L, Porter J, Coates T, Jeng M (2007). Prospective evaluation of patient-reported outcomes during treatment with deferasirox or deferoxamine for iron overload in patients with beta-thalassemia. Clin Ther.

[19] Novartis Pharmaceuticals Corporation. Exjade (deferasirox) tablets for oral suspension. Silver Spring, MD: US Department of Health and Human Services; 2013.

[20] Goldberg SL, Giardina PJ, Chirnomas D, Esposito J, Paley C, Vichinsky E (2013). The palatability and tolerability of deferasirox taken with different beverages or foods. Pediatr Blood Cancer.

[21] Rofail D, Abetz L, Viala M, Gait C, Baladi JF, Payne K (2009). Satisfaction and adherence in patients with iron overload receiving iron chelation therapy as assessed by a newly developed patient instrument. Value Health.

[22] Elalfy MS, Massoud W, Elsherif NH, Labib JH, Elalfy OM, Elaasar S (2012). A new tool for the assessment of satisfaction with iron chelation therapy (ICT-sat) for patients with beta-thalassemia major. Pediatr Blood Cancer.

[23] Barbosa CD, Balp MM, Kulich K, Germain N, Rofail D (2012). A literature review to explore the link between treatment satisfaction and adherence, compliance, and persistence. Patient Prefer Adh.

[24] Kremastinos DT, Farmakis D (2011). Iron overload cardiomyopathy in clinical practice. Circulation.

[25] Fernández-Real JM, Manco M (2014). Effects of iron overload on chronic metabolic diseases. Lancet Diab Endocrinol.

[26] Smith JA, Harré R, Van Langenhove L. Rethinking methods in psychology. London: Sage Publications; 1995.

[27] Strauss AL, Corbin JM. Basics of qualitative research: techniques and procedures for developing grounded theory. 2nd ed. Thousand Oaks, CA: Sage Publications; 1998.

[28] Willis GB. Cognitive interviewing: a tool for improving questionnaire design. Thousand Oaks, CA: Sage Publications; 2005.

[29] Coons SJ, Gwaltney CJ, Hays RD, Lundy JJ, Sloan JA, Revicki DA (2009). Recommendations on evidence needed to support measurement equivalence between electronic and paper-based patient-reported outcome (PRO) measures: ISPOR ePRO good research practices task force report. Value Health.

[30] US Department of Health and Human Services, Food and Drug Administration. Guidance for industry: patient-reported outcome measures: use in medical product development to support labeling claims. Fed Regist. 2009.

[31] Rothman M, Burke L, Erickson P, Leidy NK, Patrick DL, Petrie CD (2009). Use of existing patient-reported outcome (PRO) instruments and their modification: ISPOR good research practices for evaluating and documenting content validity for the use of existing instruments and their modification PRO task force report. Value Health.

[32] Patrick DL, Burke LB, Gwaltney CJ, Leidy NK, Martin ML, Molsen E (2011). Content validity--establishing and reporting the evidence in newly developed patient-reported outcomes (PRO) instruments for medical product evaluation: ISPOR PRO good research practices task force report: part 1--eliciting concepts for a new PRO instrument. Value Health.

[33] Patrick DL, Burke LB, Gwaltney CJ, Leidy NK, Martin ML, Molsen E (2011). Content validity--establishing and reporting the evidence in newly developed patient-reported outcomes (PRO) instruments for medical product evaluation: ISPOR PRO good research practices task force report: part 2--assessing respondent understanding. Value Health.

[34] Walton MK, Powers JH, Hobart J, Patrick D, Marquis P, Vamvakas S (2015). Clinical outcome assessments: conceptual foundation-report of the ISPOR clinical outcomes assessment - emerging good practices for outcomes research task force. Value Health.

[35] Matza LS, Patrick DL, Riley AW, Alexander JJ, Rajmil L, Pleil AM (2013). Pediatric patient-reported outcome instruments for research to support medical product labeling: report of the ISPOR PRO good research practices for the assessment of children and adolescents task force. Value Health.

[36] Yeaw J, Benner JS, Walt JG, Sian S, Smith DB (2009). Comparing adherence and persistence across 6 chronic medication classes. J Manag Care Pharm.

[37] Delea TE, Edelsberg J, Sofrygin O, Thomas SK, Baladi JF, Phatak PD (2007). Consequences and costs of noncompliance with iron chelation therapy in patients with transfusion-dependent thalassemia: a literature review. Transfusion.

[38] Squires LA, Lombardi DP, Sjostedt P, Thompson CA. A systematic literature review on the assessment of palatability and swallowability in the development of oral dosage forms for pediatric patients. Therap Innov Regul Sci. 2013;47:533–41. 10.1177/2168479013500288 30235574

[39] Alvarez O, Rodriguez-Cortes H, Robinson N, Lewis N, Pow Sang CD, Lopez-Mitnik G (2009). Adherence to deferasirox in children and adolescents with sickle cell disease during 1-year of therapy. J Pediatr Hematol Oncol.

[40] Guest G, Bunce A, Johnson L (2006). How many interviews are enough? An experiment with data saturation and variability. Field Methods.

[41] Morse JM (2000). Determining sample size. Qual Health Res.

